# Hemodynamics in the severely injured patient with significant hemorrhage

**DOI:** 10.1186/cc11058

**Published:** 2012-03-20

**Authors:** G Nardi, D Piredda, A Cossu, E Cingolani, M Cristofani, I Ghezzi

**Affiliations:** 1 S. Camillo Hospital, Roma, Italy

## Introduction

Very little is known about the hemodynamic impairments induced by trauma and severe hemorrhage. The aim of this study is to contribute to a better understanding of this topic. A recent paper has shown that about 50% of the hemorrhagic patients receive vasopressors [1] together with fluids, blood and plasma. Fluids and vasopressors are aimed to restore patients' hemodynamics; however, they might be detrimental.

## Methods

The setting was a 10-bed trauma ICU in a level 1 trauma center with a catchment population of over 2.5 million people. This is a retrospective cohort study based on the data of the ICU electronic shift. During a 24-month period (2009 and 2010), 780 patients with major trauma (ISS >15) were admitted to the hospital; 410 of them were subsequently admitted to the shock and trauma ICU. All patients with ISS >15, who had received ≥5 blood units before ICU admission, and who were submitted to semi-invasive hemodynamic monitoring (PICCO), were entered into the study.

## Results

Thirty patients (mean age 42.7 ± 17, mean 37.5 ± 12) met the study criteria. At the time of insertion of the PICCO catheter (T0) the 30 patients had already received an average of 8,760 ml fluids (3,239 ml blood, plasma and platelets, 4,870 ml crystalloids and 685 ml colloids). Systemic blood pressure, central venous pressure and heart rate at T0 were, as an average, in the normal range. Nevertheless, six patients (20%) had a Cardiac Index lower than 2.5 l/minute, and 76% had a DO_2 _significantly lower than the normal range. In the subsequent 24 hours following the information of the PICCO, these patients received, as an average, an additional 6,070 ml fluids, blood and plasma. All vasopressors were discontinued, but 40% of the patients received dobutamine. Within 24 hours (T24), oxygen transport (DO_2_) and lactate were back to the normal values in all patients but one. ICU mortality and hospital mortality were respectively 13.3% and 16%.

## Conclusion

A high percentage of the severely injured patients who received ≥5 units of PRC have a low oxygen transport at the time of ICU admission. A high percentage of them is treated with vasopressors. However, as 20% of the patients in our study had a low cardiac index in spite of a normal blood pressure and a highly positive fluid balance, vasopressors might be harmful. In our experience, hemodynamic monitoring with PICCO allowed the early recognition of inappropriate oxygen transport and a goal-directed treatment. Our data do not support the use of vasopressors to increase blood pressure in trauma patients.

## References

[B1] J Trauma 2011 71 17 19 10.1097/TA.0b013e3181be786e

